# First Record of Aff. *Plesiosuchus* sp. (Mesosuchia, Metriorhynchidae) in the Kimmeridgian of Le Havre (Normandy, North-Western France): Biometry, Profile of Locomotion, and Paleobiological Consequences

**DOI:** 10.3390/life14121595

**Published:** 2024-12-03

**Authors:** Hua Stéphane

**Affiliations:** Odyssée Paléospace, Avenue Jean Moulin, F14640 Villers-sur-Mer, France; huasteph@aol.com

**Keywords:** aff. *Plesiosuchus* sp., Kimmeridgian, Normandy, vertebrae, biometry

## Abstract

This is the first record of the biggest Metriorhynchidae, aff. *Plesiosuchus* sp. in France. The remains consist of a partial vertebral column consisting of 11 vertebrae and an ischium fragment. A new method is proposed to evaluate the individual’s size, which is estimated at 6.5 m. This method, unlike previous approaches, is based only on vertebrae and yields results that are congruent with those based on cranial remains. The state of preservation has allowed us to test the animal’s ‘profile of locomotion’ to better interpret how it moved. Concerning other metriorhynchids, the record of *Dakosaurus* in France based only on teeth must be reassessed, and the genus *Torvoneustes*, if valid, has to be distinguished from *Plesiosuchus*.

## 1. Introduction

**Figure 1:**
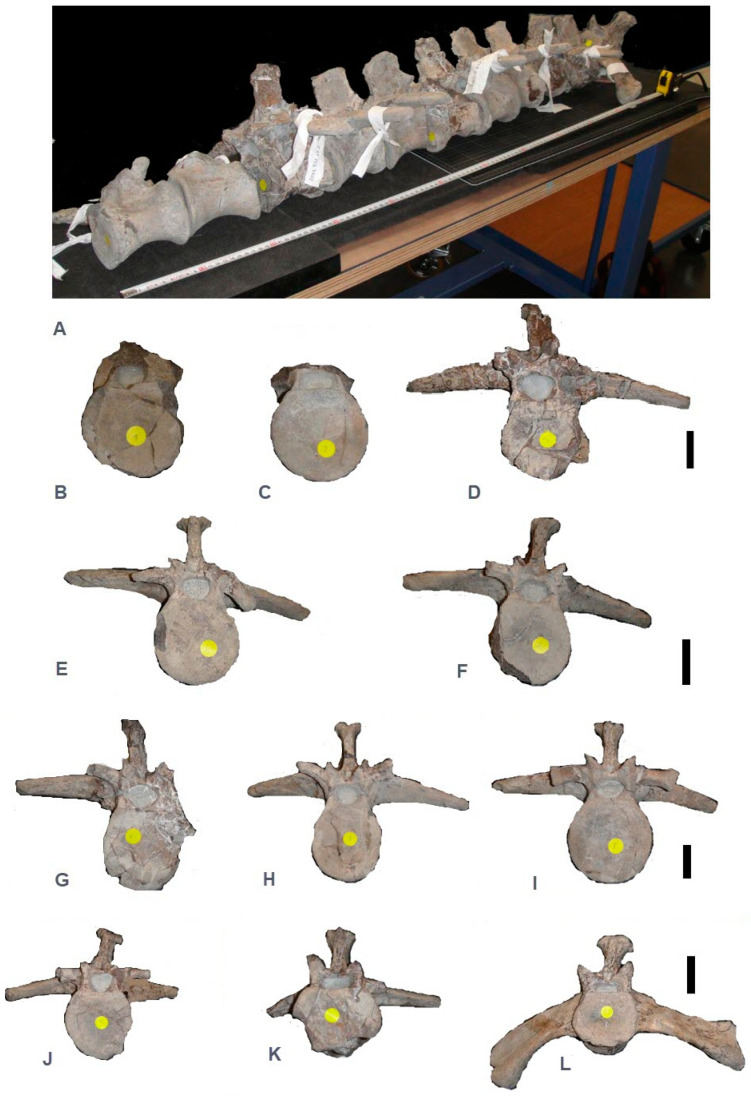
The vertebral column and all vertebrae in cranial view (scale bar = 5 cm) of aff. *Plesiosuchus* sp. (**A**) The vertebral column (MHNH 2007.5.530) shown in connection to give an idea of its impressive size (L = 117.9 cm); (**B**) 2007.5.531.9; (**C**) 2007.5.531.3; (**D**) 2007.5.531.13.1 (e); (**E**) 2007.5.531.6; (**F**) 2007.5.531.2; (**G**) 2007.5.531.13.1 (c); (**H**) 2007.5.531.1; (**I**) 2007.5.531.13.1; (**J**) 2007.5.531.13.1 (b); (**K**) 2007.5.531.13.1 (a); (**L**) 2007.5.531.13.1 (d).

**Figure 2:**
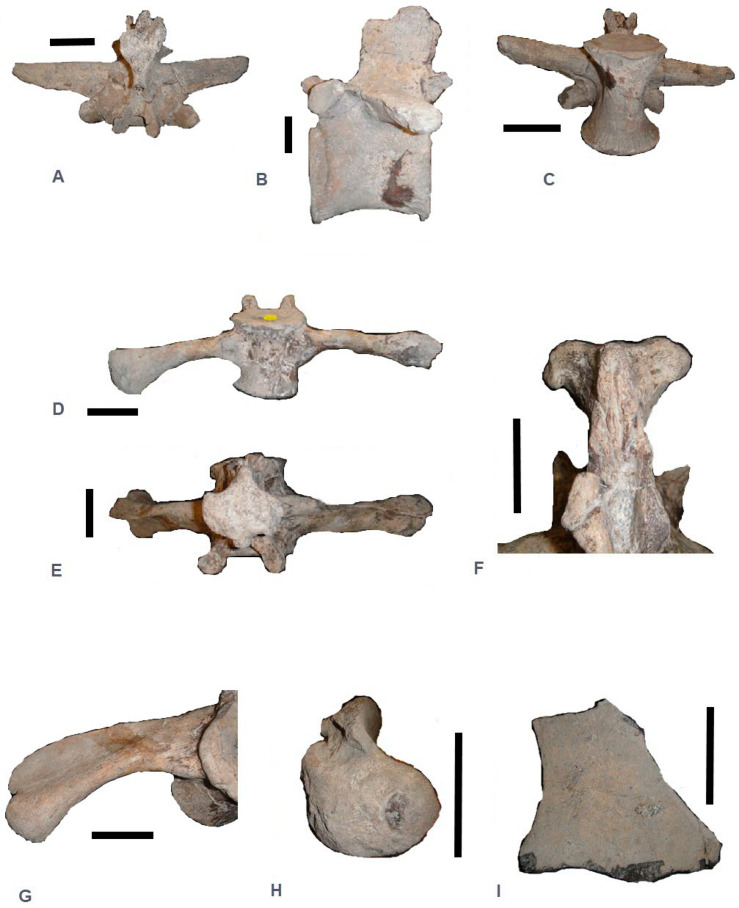
Different views of the dorsal and sacral vertebra described and the ischium. Dorsal vertebra 2007.5.531.13 in: (**A**) dorsal view; (**B**) left lateral view; (**C**) ventral view. Sacral vertebra 2007.5.531.13.1 (d): (**D**) ventral view; (**E**) dorsal view; (**F**) detail of the neural spine in posterior view showing size of muscular insertions and tuberosities. (**G**) Left sacral rib in anterior view and, (**H**) Left sacral rib in lateral view showing its “comma-shape” configuration, (**I**) fragment of left ischium in external view. Scale bars = 5 cm.

In January 1999, a block of still-embedded vertebrae and six free dorsal vertebrae was discovered by Franck Deschandol in a Kimmeridgian level at Ecqueville (Seine Maritime, north-western France). First described by Lepage et al. in 2008 [1], the mandible included in this paper was not found in association with the vertebrae. The material is kept at the Museum d’Histoire Naturelle du Havre under catalogue number MHNH 2007.5.531. The block has revealed five more nearly complete vertebrae, including a first sacral, as well as an ischium fragment from a marine crocodilian (Figure 1 and Figure 2).

Such a discovery is not surprising in this area. However, what is surprising is the size of the vertebrae: they are in fact twice the size of other specimens found in the Lower Kimmeridgian of Octeville (e.g., *Metriorhynchus* cf. *hastifer* vertebrae, in [1], p. 55). As a group, the 11 vertebrae alone are 1.17 m long (Figure 1A).

The state of preservation of the animal, besides its taxonomy, raises questions about the size of the living animal. A new biometric approached is proposed to answer to this question.

Moreover, the state of preservation of such a column allows us to gain complete knowledge of the locomotion of this animal using the method of profile of locomotion.

The partial vertebral column of this specimen was displayed as part of the exhibition *Les Crocodiliens Fossiles de Normandie* during November and December 2008, after which it became part of the collections of the Muséum d’Histoire Naturelle of Le Havre.

## 2. Geology

The location of the discovery is well identified thanks to Franck Deschandol, who discovered the specimen in January 1999 (with Gilles Lepage and Emmanuel Cacheleux) and subsequently donated it to the Museum of Le Havre (collection number 2007.5.530).

This crocodilian was found in a nodule in the middle part of the upper member of the Argiles d’Ecqueville formation, Pseudomutabilis Zone, strata XI.6 overlying the lumachelle beds, Upper Kimmeridgian [2]. Many vertebrates’ remains have already been discovered embedded in limestone nodules with crystallized calcitic hearts.

## 3. Classification

The amphicoelous ‘hourglass’ vertebrae (Figure 1) are indicative of the grade mesosuchian. In such well-known marine levels, two mesosuchian families are possible: Teleosauridae and Metriorhynchidae. Isolated dorsal vertebrae are difficult to determine, but fortunately they have quite different sacral vertebrae.

In teleosaurids, such as the Kimmeridgian *Machimosaurus mosae*, sacral ribs are more or less horizontal [3], while in metriorhynchids, they face downwards (e.g., Andrews [4]), as in this specimen (Figure 1L). On the basis of this criterion, the specimen is clearly related to Metriorhynchidae.

Compared to Middle Jurassic Callovo–Oxfordian sites like Les Vaches Noires or the Oxford Clay Formation [5,6], the French Kimmeridgian is not very rich in terms of crocodilian specimens. This family is better represented in the British deposits of the Kimmeridge Clay Formation [7,8].

Concerning the genera, four have been identified in the Kimmeridgian: *Metriorhynchus* Meyer, 1830; *Dakosaurus* Quenstedt, 1856; *Torvoneustes* Andrade et al. [9]; and *Plesiosuchus* Owen, 1884.

Clearly, the robust and huge aspect of the vertebrae allows us to eliminate the genus *Metriorhynchus* and its slender vertebrae.

The taxonomic status of the genus *Torvoneustes* is far from clear: the type species *T. carpenteri* was initially considered a species of *Metriorhynchus*, then it was assigned to *Dakosaurus* in 2008 in Young et al. [7], to *Geosaurus* in 2009 by Young et al. [8], and finally to *Torvoneustes* in 2010 by Andrade and Young [9]. A new species, *T. coryphaeus*, was erected in 2013 [10] based essentially on cranial ornamentation (such criteria must be avoided due to intraspecific or ontogenetic variation).

Such a taxonomic attribution problem within the same team (cf. previously) is not aided by the fact that *Torvoneustes* and *Plesiosuchus* are sympatric and exhibit the same size range (could the specimen described in [10] as *Torvoneustes* in fact be a younger *Plesiosuchus*?). Most of the characteristics in the description of the type species by Young et al. [11] (p. 30 and Table 1) are essentially based on differences in tooth aspect. Even though tooth pattern is important for diagnosis, it must not be the main criterion for the diagnosis: intramandibular variation exists among thalattosuchians (e.g., *Machimosaurus mosae*, in plate 2 of Hua [3]) as well as ontogenetic variation.

The genus *Dakosaurus* is known from Europe [11] to South America [12,13], mainly based on cranial remains. Even though South American fauna are rich, post-cranial remains are rare. This genus is still poorly known in the Kimmeridgian of France, even though Foffa et al. [14] indicate that it was already present in the Callovian of Villers-sur-Mer without any clear references. All the French remains are isolated ziphodont teeth or poorly preserved remains ([11]), systematically reported as *Dakosaurus* sp. (e.g., [1], p. 96), even though ziphodonty is present not only in *Dakosaurus* but also in *Plesiosuchus* and *Torvoneustes*. Taking into account that in Young et al. [10], the only differentiating characters seem to be slight tooth ornamentation with few specimens known, the presence of *Dakosaurus* in France has to be reappraised. In fact, all French remains could perfectly well belong to *Plesiosuchus*. A similar problem is observed in blunt teleosaurid teeth: when found in the Callovian, they were systematically attributed to *Steneosaurus obstusidens*, whereas when they are found in the Kimmeridgian, they were attributed to *Machimosaurus hugii* (Hua et al. [15]) until a revision was carried out by Young, Hua et al. [16].

The Kimmeridgian genus *Plesiosuchus*, with *Plesiosuchus manselii* as the type species, is represented by a mandible and an incomplete skull. It was initially described by Owen in 1868 [17] as a pliosaur. In 1869, Eudes-Deslongchamps [18] demonstrated that the specimen was of metriorhynchid nature. Later authors confirmed this attribution, apart from Vignaud [12], who interpreted the specimen as *Machimosaurus mosae.* In 2012, Young et al. [11] clearly demonstrated the existence of *P. manselii* with an exhaustive description and diagnosis. In the absence of skull remains, as is the case here, all determinations must be considered tentative.

The size of the vertebrae and their shape, with their impressive tuberosities on the neural spine, are unknown for a specimen of genus *Metriorhynchus* of the same level [1], and for this reason it cannot be attributed to *Metriorhynchus*. *Plesiosuchus* vertebrae are unknown, but estimated size and stratigraphic level could make this genus as a good candidate.

A generic attribution would be too speculative, so this specimen is referred to as aff. *Plesiosuchus* sp.

The importance of this specimen is that it is the first relatively complete vertebral column referable to this genus and the first from French deposits.

As such, based on this discussion, the classification of this specimen is considered to be as follows:

Superorder Crocodylomorpha Hay, 1930 (*Sensu* Nesbitt, 2011) [19];

Order Mesoeucrocodylia Whestone and Whybrow, 1983 [20];

Suborder Thalattosuchia Fraas, 1901 [21];

Family Metriorhynchidae Fitzinger, 1843 [22];

Genus aff. *Plesiosuchus* Owen, 1884 (*sensu* Young et al. 2012 [11]).

## 4. Description

Only 3 out of 11 vertebrae will be described in detail to highlight their typical shape: a dorsal, a lumbar, and the sacral.

All measurements are provided in Table 1.

The bauplan of these vertebrae do not differ from other Metriorhynchidae for the Callovian by Andrews [4] or for Oxfordian by Lepage et al. [1] (pp. 54–56) for the genus *Metriorhynchus*, apart from their huge and robust aspect.

The vertebra 2007.5.531.13 (Figure 1I and Figure 2A–C) described here is considered to be a mid-dorsal vertebra. The centrum has an hourglass shape, like those of all mesosuchians, with the height of the anterior face being larger than the posterior one.

The articular ends of the centrum are moderately concave, with the vertical diameter being the greatest, and are slightly flattened at the level of the neural canal. Both faces of the centrum are slightly concave, and curiously for such an animal, no crenulations from muscular insertions are visible in the lateral view (Figure 2B). Unfortunately, no other such preserved metriorhynchid vertebral columns from the Kimmeridgian are known, but this needed to be described in case of a new discovery.

The most striking feature of these dorsal vertebrae is the robustness of the parapophysis process, which has a rounded section that is upward-oriented and is separated from the diapophysis by a distance of 3 cm. The articular surface of the parapophysis, with a diameter of nearly 1.5 cm (Figure 2A), indicates the robustness of the ribs.

The diaphyses are massive with the prezygapophyses included. The articular surface of the prezygapophyses is piriform, flat, and measures approximately 2 × 1 cm.

The diaphyses appear to be horizontal with a blade-like section in the anterior vertebrae and are more rounded and downward-oriented in the last dorsal vertebrae. In the dorsal view, the anterior border of the diaphysis process is convex and the posterior border is linear, finishing before the level of the anterior face of the centrum. The neural spines are developed in the last two-thirds of the centrum length and are relatively elevated with strong lateral tuberosity for muscular insertion, starting just after the posterior border of the parapophysis in the dorsal view. Such tuberosities are present and visible on skeletons of *Metriorhynchus*, but here it is very pronounced due to the size of the animal. Rectangular in lateral view, they overhang the posterior face of the centrum (Figure 2A).

The postzygapophyses are elongated, slightly convex, piriform in shape, and measure 2 × 1 cm.

In the dorsal area, it is interesting to highlight the difficulty of determining the number of lumbar vertebrae in metriorhynchids: Andrews [4] observed one or two lumbars; on the *Me.* cf. *superciliosus* described in Hua et al. [6], only one lumbar is identified with certainty; in the specimen described from the MNHN of Paris (MNHN 1908-6, pers. obs.), only one is visible; likewise, in this specimen, only one lumbar vertebra could be identified.

The vertebra 2007.5.531.13.1 (a) (Figure 1K) is interpreted as dorsal and more especially lumbar due to the smaller transverse process, which is downward-facing and not totally transversal like the others. Moreover, the paraphysis is fused with the diapophysis with a regressed parapophysis head of less than one cm. The cross section of the parapophysis is no more rounded than in the other vertebrae. The prezygapophysis still appears strong, with an angle of nearly 94°, and is totally independent from the diapophysis. The neural spine is shorter than the height of the centrum, but always bears strong transverse tuberosities due to muscular insertions. Despite its less well-preserved state, the centrum appears more circular than in the dorsal vertebrae.

In their general appearance, no dorsal vertebrae differ significantly from those described for another species of contemporaneous thalattosuchians, the teleosaurid *Machimosaurus mosae* [3]. The only difference is in the muscular tuberosities on the neural spines, which are much more developed on this specimen, indicating a more mobile trunk than is the case for *Machimosaurus*. This result is not surprising, as the trunk of *M. mosae* must have been more rigid with two osteodermal shields, one ventral and one dorsal.

The first sacral vertebra, 2007.5.531.13.1 (d) (Figure 1L and Figure 2D–H), is complete and is typical of metriorhynchids, with sacral ribs facing downward and not transversally (e.g., as in *M. mosae*, plate 3 in [5]). The centrum is similar to that of the previous lumbar vertebrae. It is more massive, nearly quadrangular, with a section that is more transverse than vertical (the reverse for the dorsals). The prezygapophyses are well developed, with a surface measuring more than 2 × 1 cm, and are separated from the sacral ribs. In section, the ribs are in a comma-shape configuration (Figure 2H): a ventral border convex with a thin dorsal border. The connection with the ilium is 4 × 4 cm, below the level of the centrum.

The sacral ribs are inserted on the full height of the centrum, constricting at the middle to expand vertically for the insertion of the ilium over a height of more than 5 cm.

As for the lumbar vertebra, the neural spine is shorter than the height of the centrum, still with very pronounced transverse muscular tuberosities that protrude almost 2 cm beyond the surface of the neural spine (Figure 2F). The postzygapophysis appears weak in comparison to the size of the prezygapophysis.

The ischium is partial (Figure 2I), consisting of just a portion of the distal blade. This bone is thin (5 mm), showing slight convexity, as in all other metriorhynchids [4].

## 5. Size Estimation

Based on the work of Young et al. in [23], it is possible to estimate the size of an individual according to the size of the skull and the femur.

In this case, this was not possible because only eleven articulated vertebrae are present to work on. To estimate the size of this metriorhynchid, three nearly complete skeletons with articulated vertebrae that are well-preserved and not too distorted were used, as follows.

*Me.* cf. *superciliosus* FBS2012.4.67.80 described in Hua et al. [6], Callovian of the Vaches Noires, well preserved without the skull, estimated size 2.9 m.

*Me.* cf. *superciliosus* MPV 2010.3.610 described in Le Mort et al. [5], Callovian of the Vaches Noires, well preserved with partial skull, estimated size 2.5 m.

*Me. superciliosus* MNHN1908.6 exposed in the gallery from the Callovian of Peterborough, slightly crushed but complete, estimated size 1.90 m.

The skeleton of the teleosaurid, *Machimosaurus mosae* BHN2R1100, from the Kimmeridgian of Boulogne sur Mer, which is complete and well preserved with an estimated size of 5 m, has been also used.

The first surprising finding is that all the *Me. superciliosus* specimens used here are smaller (no more than 2.9 m) than the specimens measured in the study of Young et al. [8] (3 m to 3.5 m), also based on complete skeletons (but crushed). Furthermore, in estimating the size of a mesosuchian, an additional difficulty is the size of the intervertebral discs. This is always difficult to estimate, but in some complete skeletons, like the *Geosaurus suevicus* in the frontispiece of Andrews [4], these discs seem not to be too thick.

Young et al. [23] based their estimations on the length of the skull and the femur.

The approach here is totally different and independent based only on the size of vertebrae. By this method, the results could be compared if they are congruent.

With this specimen, two criteria have been used: the first one with all of them and the second one only using the first sacral vertebra.

The estimations are based on two criteria, detailed in Table 2.

The calculation is based on a basic rule of three, with measurements taken directly on the specimen.

Criterion 1: Total length of the 10 last dorsals + first sacral vs. total length of the skeleton.

Criterion 2: Length of the first sacral (not crushed) vs. total length of the skeleton.

Firstly, here the size of the adult specimens varies between 1.9 m and 2.9 m, whereas for Young [18], they vary from 3 m to 3.5 m (on crushed fossils). On complete skeletons of adults of the same species, ±1 m of variation for adults of the same species appears coherent with what could be seen in the wild for living crocodylians of the same species of adults of different age.

Secondly, results obtained through comparison with *Machimosaurus mosae* show that isometric rules are valid only per family (even close like teleosaurids): the results provided by *M. mosae* are too different to be taken into account. The results here underline it.

According to the calculation of criterion 1, a total size of 6.5 m appears close to the result of Young et al. [11] (with a length of 6.8 m) using other isometric rules (based on skull and femora), whereas here only vertebrae were used.

As already mentioned, ±1 m could appear for adults of the same species of various age, so this result appears congruent with the result of Young et al. [23] and can be taken to indicate the size of this specimen. Moreover, this study shows that results obtained using only the length of the vertebrae could be useful for estimating the length of such crocodilians.

The calculation based only on criterion 2, restricted to the first sacral vertebra, seems congruent and could be useful to estimate size on the basis of only one vertebra, which has never been done before.

Nevertheless, the results obtained are compatible with the results of Young et al. [11] (measured on a totally different biometric basis, confirming both results (this paper and the study of Young et al. [23])). *Plesiosuchus* is considered the giant of the Metriorhynchidae. This is not so surprising, for generally speaking, the marine crocodilian, including its living relative—*C. porosus*, the saltwater crocodilian—is known to reach a length of 7 m [24].

## 6. Profile of Locomotion

First used by Hua [25], the technique applied here is based on the systematic angle of aperture of the zygapophyses across the vertebral column. This method of measurement was developed in order to compare dominant movements according to anatomic position between various taxa. Another advantage of this measurement is that it is independent of the thickness of the intervertebral disc and the intervertebral articulation, especially among crocodilians.

This is an advantage because from a purely anatomical point of view, and regardless of the classification used, two totally different kinds of articulations have historically existed in crocodilians: the amphicoelous vertebrae of the Protosuchians and Mesosuchians [26] and the procoelous vertebrae of the Eusuchians, i.e., ‘flat’ intervertebral articulation on the one hand and ‘ball and socket’ on the other. The latter development must have greatly increased the vertebral flexibility for eusuchians, regardless of taxon (Buffetaut [26]).

An additional difficulty in interpreting the axial mobility of the metriorhynchids is the fact that they were the only family without dorsal osteoderms, whereas some contemporaneous teleosaurids had three osteodermal shields (one dorsal and two ventral ones).

Therefore, when choosing between intervertebral joints or osteodermal shields to estimate the body mobility of such animals, we have to be careful to compare similar anatomic configuration.

Molnar et al. [27] tried to estimate the flexibility of various crocodilian taxa, including modern crocodilians and thalattosuchians, based in part on very interesting experimental results on living specimens. Nevertheless, these results must be tempered by the ball-and-socket intervertebral joint of the Eusuchians versus the acoelous articulation of the fossil taxa studied in this paper.

**Figure 3:**
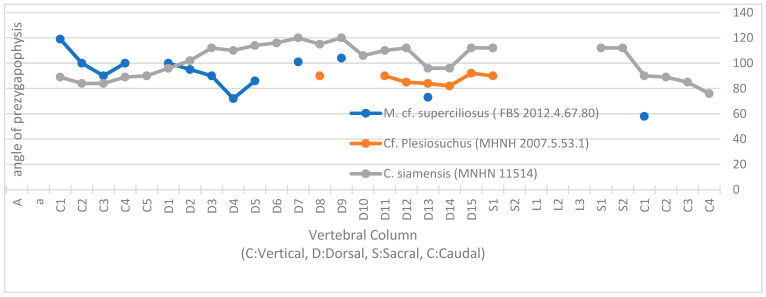
Aperture angle of prezygapophyses as a proxy for locomotion profile in a living eusuchian (*Crocodylus siamensis*) and a sub-complete *Metriorhynchus superciliosus* (Thalattosuchia, Metriorhynchidae) and aff. *Plesiosuchus*. Abbreviations: FBS, Fabrique des Savoirs d’Elbeuf; MNHN, Muséum d’Histoire Naturelle de Paris; MHNH, Muséum d’Histoire Naturelle du Havre; C, cervical; D, dorsal; S, sacral; C, caudal.

The locomotion profile of the specimen (in orange, Figure 3) is like that of other Metriorhynchidae for the thoracic region, where sharp angles indicate dominant vertical movements, whereas modern crocodilians undulate laterally when swimming.

Interestingly, the angle of the aperture tends to exceed 90°, which is more than was observed for the other measured metriorhynchids by Hua [25]. Their trunk would thus have been flexible, allowing a greater degree of lateral movement than their close relatives like *Metriorhynchus*, while still maintaining greater rigidity for more efficient swimming than living crocodilians.

This anatomical polyvalency could indicate a behaviour different from the long-snouted and ichthyophageous *Me. superciliosus* noted by Hua [28]: the largest members of this family could have been more microphagous, as suggested by Molnar et al. [27]. For measurements made, all skeletons used were adults (neurocentral suture totally invisible), so ontogenetic variation can be discounted in the interpretation of the results.

This study underlines the fact that isolated vertebral column can be rich in information from the taxonomic point of view and also from a paleobiological point of view (biometric rules and locomotion profile).

## 7. Discussion: Thalattosuchians from the French Kimmeridgian

This partial skeleton, the first aff. *Plesiosuchus* to be confirmed in France, gives us a better understanding of thalattosuchian fauna during the Kimmeridgian.

The Kimmeridgian crocodilian fauna in France bear the largest thalattosuchian taxa: *Machimosaurus*, around 5–6 m in length for the Teleosauridae and *Plesiosuchus* and approximately 6.5 m in length for the Metriorhynchidae. More precisely, during the Callovian, both longirostrine (e.g., *Me. superciliosus*) and brevirostrine species (e.g., *Me. brachyrhynchus*)—Hua, 2008 in [1]—were about moderate size (around 2.5–3 m) and became more massive like *Plesiosuchus*, with double the size being observed for the first time during the Kimmeridgian in France.

It should be noted that this doubling phenomenon (increasing size combined with mesorostrine vs. longirostrine bipolarization) is the same for both members of the thalattosuchian group, Teleosauridae and Metriorhynchidae. The reason for this must be found with other coexisting epipelagic marine reptiles, like the other marine macropredators, the plesiosaurids, which were in regression during the Upper Jurassic, as described by Godefroit [29].

Besides size, the skull of such crocodilians converges in terms of shape towards the sauropterygian skull. This initially even misled Owen in his identification, especially because of the presence of a huge mandible without mandibular fenestrae, a unique feature among crocodilians.

The crocodilian skull is always a compromise between rigidity (to withstand torsion forces during the killing of prey) and lightness, phenomena underlined by Langston [30].

The closing of the mandibular fenestrae may have been a response to lightning caused by porosity of the cranial bones described by Hua and De Buffrénil [31] by increasing the rigidity of the skull. This modification would have led the skull shape to converge with that of the sauropterygians, explaining the initial mistake of Owen in 17].

The genus *Torvoneustes* must be reassessed to be differentiated with autapomorphic characters from the genus *Plesiosuchus*, given that for the moment, they cannot be safely distinguished.

These remains are from one of the largest metriorhynchids (approximatively 6.5 m in length) ever recorded in France. In addition, part of the axial skeleton survives in connection, the first such occurrence for the genus *Plesiosuchus.*

A new methodology has been used to estimate the animal’s length, and the result seems to be corroborated by biometric measurements based on other criteria.

## Figures and Tables

**Table 1:** Measurements of the 11 vertebrae of MNHN 2007.5.531. * Estimated measurement, total length, 117.9 cm.

Vertebrae	Anatomical Position	Inventory Number	Length of Centrum in Mid Ventral Line (cm)	Width of Anterior Face of Centrum (cm)	Height of Anterior Face of Centrum (cm)	Width of Posterior Face of Centrum (cm)	Height of Posterior Face of Centrum (cm)	Total Width	“Total Height (Neural Spine + Centrum)”	pre_Alpha	Post Alpha
Dorsal	6	2007.5.531.9	12.0	11.5	11.0	12.0	11	KO	KO	90	KO
D.	7	2007.5.531.3	12.5	12.0	11.5	10.0	10	KO	KO	KO	KO
D.	8	2007.5.531.13.1 (e)	10 *	8 *	8 *	10 *	10 *	34.5	KO	KO	KO
D.	9	2007.5.531.6	10.0	10.0	10.5	10.0	9	32	2105	90 *	75
D.	10	2007.5.531.2	10.5 *	10 *	10.0	11.0	10 *	27.5	22	85	55
D.	11	2007.5.531.13.1 (c)	11.0	10.2	10*	11 *	10	26 *	24	84	KO
D.	12	2007.5.531.1	11.0	9.9	10.3	10.0	10.8	32.2	23	82	60
D.	13	2007.5.531.13.1	10.0	11.0	11.0	11.0	10	28.5	22.5	92	60
D. (Lumbar 1)	14	2007.5.531.13.1 (b)	9.5	11.0	11.0	10.0	10	28 *	23	KO	90 *
D. (Lumbar 2)	15	2007.5.531.13.1 (a)	11 *	12.0	10 *	10 *	8 *	28 *	20	94 *	KO
Sacral 1	16	2007.5.531.13.1 (d)	10.4	11.5	8.0	8.1	8.4	37.6	18	90	84

**Table 2:** Calculation of the total length of the specimen Tl according to the two criterias (related Cvert or just the first sacral) with different close relative specimens.

	*M. superciliosus*	*M.* cf. *superciliosus*	*M.* cf. *superciliosus*	*Machimosaurus mosae*	Aff. *Plesiosuchus*
	MNHN 1908-6	MPV 2010.3.610	FBS 2012.4.67.80	BHN2R1100	MHNH 2007.5.531
Crit1.: Total length of the (10D+1S) = Cvert (in cm)	34.1	45	53.5	68.4	117.9
Crit2.: Length of the S1 = LS1 (in cm)	3	3.9	4.5	5.1	10.4
Total length of the specimen (in cm) = Tl	240	250	290	500	Tl
size Aff. *Plesiosuchus* (Tl in cm) according to Crit 1 = f(specimen)	656.9	655	639.1	861.8	
size Aff. *Plesiosuchus* (Tl in cm) according to crit 2 = f(specimen)	658.7	666.7	670.2	1019.6	

Abbreviations: BHN, Boulogne sur Mer Musée d’Histoire Naturelle; FBS, Fabrique des Savoirs d’Elbeuf; MNHN, Muséum d’Histoire Naturelle de Paris; MHNH, Muséum d’Histoire Naturelle du Havre; MPV, Musée Paleospace de Villers.

## Data Availability

Enclosed in the study.

## References

[1] Lepage Y., Buffetaut E., Hua S., Martin J.E., Tabouelle J. (2008). Catalogue descriptif, anatomique, géologique et historique des fossiles présentés à l’exposition “Les Crocodiliens fossiles de Normandie” (6 novembre–14 décembre 2008). Bull. Soc. Geol. Normandie.

[2] Samson Y., Lepage G., Hantzpergue P., Guyader J., Saint-Germain S.M.M., Baudin F., Bignot G. (1996). Révision lithostratigraphiqne et biostratigraphique du Kimméridgien de la région havraise (Normandie, France). Bull. Soc. Géol. Fr..

[3] Hua S. (1999). The Crocodilian *Machimosaurus mosae* (Thalattosuchia, Teleosauridae) from the Kimmeridgian of the Boulonnais (Pas de Calais, France). Palaeontogr. Abt. A Palaozool..

[4] Andrews C.W. (1913). A Descriptive Catalogue of Marine Reptiles of the Oxford Clay.

[5] Le Mort J., Martin J.E., Picot L., Hua S. (2022). First description of the most complete *Metriorhynchus* cf. *superciliosus* (Thalattosuchia) specimen from the Callovian of the Vaches-Noires cliffs (Normandy, France) and limitations in the classification of Metriorhynchidae. Ann. Pal. Paris.

[6] Hua S., Liston J., Tabouelle J. (2024). The Diet of *Metriorhynchus* (Thalattosuchia, Metriorhynchidae): Additional Discoveries and Paleoecological Implications. Foss. Stud..

[7] Wilkinson L.E., Young M.T., Benton M.T. (2008). A new metriorhynchid crocodile (Mesoeucrocodylia, Thalattosuchia) from the Kimmeridgian (Upper Jurassic) of Wiltshire, UK. Paleontology.

[8] Young M.T., De Andrade M.B. (2009). What is *Geosaurus*? Redescription of *Geosaurus giganteus* (Thalattosuchia: Metriorhynchidae) from the Upper Jurassic of Bayern, Germany. Zool. J. Linn. Soc..

[9] Andrade M.B., Young M.T., Desojo J., Brusatte S.L. (2010). The evolution of extreme hypercarnivory in Metriorhynchidae (Mesoeucrocodylia: Thalattosuchia) based on evidence from microscopic denticle morphology. J. Vert. Pal..

[10] Young M.T., Andrade M.B., Brusatte S.L., Sakamoto M., Liston J.J. (2013). The oldest known metriorhynchid super-predator: A new genus and species from the Middle Jurassic of England, with implications for serration and mandibular evolution in predacious clades. J. Syst. Pal..

[11] Young M.T., Brusatte S.L., Brandalise de Andrade M., Desojo J.B., Beatty B.L., Steel L., Fernandez S., Sakamoto M., Ruiz-Omenaca J.I. (2012). The cranial osteology and feeding ecology of the Metriorhynchid Crocodylomorph genera *Dakosaurus* and *Plesiosuchus* from the late Jurassic of Europe. PLoS ONE.

[12] Vignaud P. (1995). Les Thalattosuchia, Crocodiles Marins du Mésozoique: Systématique Phylogénétique, Paléoécologie, Biochronologie et Implications Paléogéographiques. Ph.D. Thesis.

[13] Fernandez M.S., Herrerra Y. (2021). Active airflow of the paranasal sinuses in extinct crocodyliforms: Evidence from a natural cast of the thalattosuchian *Dakosaurus andiniensis*. Anat. Rec..

[14] Foffa D., Young M.T., Brussate S.L., Graham M.R., Steel L. (2017). A new metriorhynchid crocodylomorph from the Oxford Clay Formation (Middle Jurassic) of England, with implications for the origin and diversification of Geosaurini. J. Syst. Palaeontol..

[15] Hua S., Vignaud P., Pennetier E., Pennetier G. (1994). Un squelette de *S. obtusidens* Andrews, 1909 dans le Callovien de Villers sur Mer (Calvados, France) et le problème de la définition des Teleosauridae à dents obtuse. C. R. Acad. Sci. Paris.

[16] Young M.T., Hua S., Steel L., Foffa D., Brusatte S.L., Thüring S., Mateus O., Ruiz-Omeñaca J.I., Havlik P., Lepage Y. (2014). Revision of the Late Jurassic teleosaurid genus *Machimosaurus* (Crocodylomorpha, Thalattosuchia). R. Soc. Open Sci..

[17] Owen R. (1868). Monography on the British Fossil Reptilian from the Kimmeridge Clay—Sauropterygia.

[18] Eudes-Deslongchamps A. (1869). Prodrôme des Téléosauriens du Calvados. Notes paléontologiques 1867–1869.

[19] Nesbitt S.J. (2011). The Early Evolution of Archosaurs: Relationships and the Origin of Major Clades. Bull. Amer. Mus. Nat. Hist..

[20] Whetstone K.N., Whybrow P.J. (1983). A “Cursorial” Crocodilian from the Triassic of Lesotho (Basutoland). South. Afr. Occas. Pap. Mus. Nat. Hist. Univ. Kans..

[21] Fraas E. (1901). Die Meerkrokodile (*Thalattosuchia* n. g.) eine neue Sauriergruppe der Juraformation. Jahresh. Des Ver. Für Vaterländische Naturkunde Württemberg.

[22] Fitzinger L.J.F.J. (1843). Systema Reptilium.

[23] Young M.T., Bell M.A., De Andrade M.B., Brusatte S.L. (2011). Body size estimation and evolution in metriorhynchid crocodylomorphs: Implications for species diversification and niche partitioning. Zool. J. Linn. Soc..

[24] Wikipedia 2014—List of Largest Reptiles. https://en.wikipedia.org/w/index.php?title=List_of_largest_reptiles&oldid=1221630486.

[25] Hua S. (2003). Locomotion in marine mesosuchians (Crocodylia): The contribution of the “locomotion profiles”. Neues Jahrb. Für Geol. Und Paläontologie-Abh..

[26] Buffetaut E. (1982). Radiation, Evolutive Paléoécologie et biogéographie des crocodiliens mésosuchiens. Mémoires De La Société Géologique De Fr..

[27] Molnar J.L., Pierce S.E., Bhullar B.A.S., Turner A.H., Hutchinson J.R. (2015). Morphological and functional changes in the vertebral column with increasing aquatic adaptation in crocodylomorphs. R. Soc. Open Sci..

[28] Hua S. (1994). Hydrodynamique et modalités d’allègement chez Metriorhynchus superciliosus (Crocodylia, Thalattosuchia): Implications paléoécologiques. Neues Jahrb. Geol. Palaontol. Abh..

[29] Godefroit P. (1994). Les Reptiles Marins du Toarcien (Jurassique Inférieur) Belgo-Luxembourgeois.

[30] Langston W., Gans C., Bellairs A., Parsons T.S. (1973). The crocodilian skull in historical perspective. Biology of the Reptilia.

[31] Hua S., De Buffrenil V. (1996). Histology of the Thalattosuchia as a clue of the interpretation of functional adaptations in the Thalattosuchian (Reptilia, Crocodylia). J. Vert. Pal..

